# How New Yorkers Are Housed

**Published:** 1889-08

**Authors:** 


### HALL'S JOURNAL OF HEALTH

TRUTH DEMANDS NO SACRIFICE; ERROR CAN MAKE NONE.

VOL. 36: AUGUST, 1889. No. 8.

### HOW NEW YORKERS ARE HOUSED.

Families residing in the great City of New York are variously housed and domiciled. Very many occupy an entire dwelling of from ten to twenty rooms, for the most part as tenants, but by far the greater number, though well-to-do, live in flats, a comparatively late introduction.

A flat consists of the requisite accommodations for the family on a single floor. They are variously constructed and usually contain from seven to ten rooms, including kitchen and bath room. Many of the better class are rented completely furnished. Each story has its complement for one or more families independently of the others. There are often as many as eight or ten stories or series of flats. The more modern and costly ones are steam heated throughout, with separate passenger and truck and baggage elevators which respond to the touch of the electric button. All the household supplies are received by an attendant in the basement, and elevated without confusion, by steam power, warned by a timely signal, and the household refuse and offal are in like manner sent down, to be seen no more.

The next in order, in a descending scale, is the apartment house, usually occupying a twenty-five foot lot with a narrow hall running through the middle, and two sets of apartments on either hand, consisting of from four to five rooms. The upper floors are substantially the same, the chief difference being that above the ground floor the hall space is thrown into the two front and back rooms, the entrances to which are at an obtuse angle.

As a general thing, all flat and apartment houses have separate coal depositories in the basement or cellar for each tenant.

After apartments come tenement houses of which no general description can be given, as they follow no general plan in construction and arrangment. Many of them are simply houses which have fallen into disuse by prior occupants, owing to neighborhood and other changes, and by a kind of enforced degeneracy, been given over to the only uses which they can be made to serve. There are whole districts of these, some exclusively Italian, some Irish, some colored, and again we are glad to say some of the better class, German.

It is needless to say, that in hundreds of these lower order houses, vast populations are huddled together more like pigs in a pen than as becomes intelligent human beings, and it is suprising that the health roll of these neighborhoods is as clear as the reports indica e.

